# A tree’s quest for light—optimal height and diameter growth under a shading canopy

**DOI:** 10.1093/treephys/tpaa110

**Published:** 2020-09-02

**Authors:** Peter Fransson, Åke Brännström, Oskar Franklin

**Affiliations:** Department of Mathematics and Mathematical Statistics, Umeå University, Linneaus väg 49, Umeå, SE-901 87, Sweden; Department of Forest Ecology and Management, Swedish University of Agricultural Sciences, Skogsmarksgränd 17, SE-901 83 Umeå, Sweden; Department of Mathematics and Mathematical Statistics, Umeå University, Linneaus väg 49, Umeå, SE-901 87, Sweden; International Institute for Applied Systems Analysis, Schlossplatz 1, Laxenburg, A-2361, Austria; International Institute for Applied Systems Analysis, Schlossplatz 1, Laxenburg, A-2361, Austria; Department of Forest Ecology and Management, Swedish University of Agricultural Sciences, Skogsmarksgränd 17, SE-901 83 Umeå, Sweden

**Keywords:** allocation, growth strategy, life history, optimal control, tree

## Abstract

For trees in forests, striving for light is matter of life and death, either by growing taller toward brighter conditions or by expanding the crown to capture more of the available light. Here, we present a mechanistic model for the development path of stem height and crown size, accounting for light capture and growth, as well as mortality risk. We determine the optimal growth path among all possible trajectories using dynamic programming. The optimal growth path follows a sequence of distinct phases: (i) initial crown size expansion, (ii) stem height growth toward the canopy, (iii) final expansion of the crown in the canopy and (iv) seed production without further increase in size. The transition points between these phases can be optimized by maximizing fitness, defined as expected lifetime reproductive production. The results imply that to reach the canopy in an optimal way, trees must consider the full profile of expected increasing light levels toward the canopy. A shortsighted maximization of growth based on initial light conditions can result in arrested height growth, preventing the tree from reaching the canopy. The previous result can explain canopy stratification, and why canopy species often get stuck at a certain size under a shading canopy. The model explains why trees with lower wood density have a larger diameter at a given tree height and grow taller than trees with higher wood density. The model can be used to implement plasticity in height versus diameter growth in individual-based vegetation and forestry models.

## Introduction

In forests, a tree’s growth strategy is to a large extent determined by a struggle for light, either by growing taller toward brighter conditions or by expanding leaf area or crown size to capture more of the available light. In addition, the tree’s growth strategy is not only important for the tree itself, but due to the dominant ecological role of trees it has consequences for the whole forest ecosystem. However, despite the critical importance of this height versus crown size strategy for trees, to our knowledge, a general model for the optimal growth strategy under a shading canopy has not yet been presented.

A plethora of growth and allocation models exists (Franklin et al. 2012) ranging from the most simple fixed allometric relationships to game-theoretic optimality models of even-aged stands (Mäkelä 1985, King 1993), and sophisticated mechanistic models of tree evolution (Falster et al. 2017). However, while the optimality models only apply to even-aged stands of identical trees, the evolutionary models do not account for plasticity of the growth path in response to a variable light environment. Thus, none of the existing models addresses the problem that trees growing under established canopies face: how can the growth path of an individual tree be optimized in terms of height, crown size and reproduction under the prevailing light environment?

This problem is a specific case of the more general problem of optimal growth allocation, which has long been an intense area of research in plant ecophysiology, and which has been addressed using many different approaches (Franklin et al. 2012). Perrin (1992) investigated, for organisms in general, how resources should be optimally allocated when accounting for both growth and mortality, showing that the ratio between production and mortality (hereafter we denote this ratio as *P*:*m*) plays an important role for optimal allocation. Plants should invest all resources into the organ that gives the highest return in terms of this ratio. Only when two or more organs give the same increase in the ratio, should simultaneous allocation be carried out. However, in this study, plants were assumed to grow in constant full light, which contrasts with conditions in many forests, where the light availability increases with height. Thus, it is unclear whether the local-growth optimization strategy to maximize *P*:*m* under current conditions (Perrin 1992) is still valid. Instead it may pay to overinvest in height growth initially, in order to reach better light conditions more quickly. To address this problem, it is necessary to optimize the allocation with respect not only to current conditions but also to the expected future conditions.

**Figure 1.**
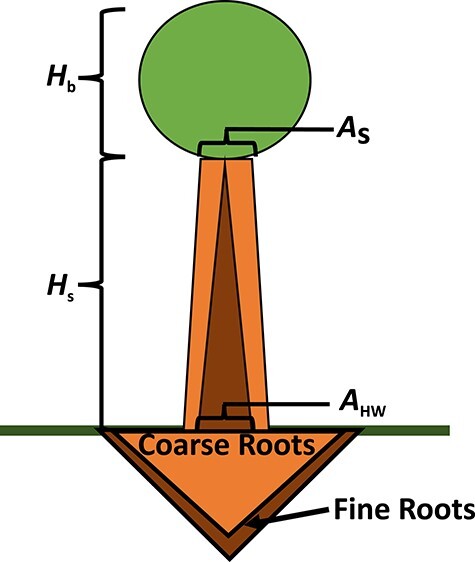
The light environment (a) is defined by the three properties: (1) the light level at initial stem height *Q*_0_, (2) the maximum light gradient *dQ*_max_ and (3) the height at maximum light gradient *H*_dQmax_. The light level increases with the height of the tree (which is determined by the stem height and crown size). (b) The corresponding light gradient (derivative).

In this study we model optimal growth paths in terms of height, crown size and seed production, based on maximization of lifetime fitness using a mechanistic model of tree growth and mortality. We address the following questions: can optimal growth paths be determined based on optimization with respect to the local environment only (as in previous optimal allocation models), and if not, what does the optimal path look like? Can the model provide an explanation for observed tree height growth strategies in forests, and differences between fast strategies with low wood density and slow strategies with high wood density?

## Theory and model

### Light environment

We assume that the light environment that a tree experiences varies only with its total height *H*_tot_, which is the sum of the stem height *H*_s_ and the crown height *H*_b_. The light level is measured relative to the above canopy level, assuming values between 0 and 1. The light profile (*Q*) is described by a sigmoid function of tree height *H*_tot_ as illustrated in Figure 1 (equation A. 2 in Appendix A available as Supplementary Data at *Tree Physiology* Online). The choice of functional form is motivated by the shape of observed height-light level curves in a rain forest (Poorter et al. 2005). The shape of the light profile is determined by three properties: (1) the light level at initial tree height *Q*_0_, (2) the maximum light gradient, *dQ*_max_ and (3) the height at maximum light gradient, *H*_dQmax_. For a detailed description of the light profile see Appendix A available as Supplementary Data at *Tree Physiology* Online. The different light environments used in the simulations were created by assigning different values for ${H}_{\mathrm{dQmax}}$, ${dQ}_{\mathrm{max}}$ and ${Q}_0$ in the ranges 5 to 30 m, 0.07 to 0.36 m^−1^ and 0.23 to 0.40, respectively.

**Table 1:** List of symbol definitions, units and default parameter values used in the main text.

Symbol	Unit	Value	Description	Source(s) and motivation
Tree variables				
*P*	kg year^−1^		Net production (dry matter)	
*W*_f_, *W*_s_, *W*_b_, *W*_cr_	kg		Dry matter weight^*^	
*A*_s_, *A*_b_	m^2^		Sapwood area^*^	
*A*_HW_	m^2^		Heartwood area	
*H*_s,_ *H*_b_	m		Height^*^	
Tree parameters				
*r*_G_	−	0.25	Growth respiration constant	Mäkelä (1986)
*K*_p_	Year^−1^	4	Maximum gross production rate	Mäkelä (1986)
*K*_f_	Year^−1^	0.5	Cost rate for foliage	Mäkelä and Valentine (2001)
*K*_s_/*K*_b_/*K*_cr_	Year^−1^	0.03	Cost rate for sapwood	Mäkelä and Valentine (2001)
*K*_fr_	Year^−1^	0.5	Cost rate for fine roots	Mäkelä and Valentine (2001)
η_b,1_	m^−2ηb,2 + 1^	0.5	Power law constant relating crown height to sapwood area	Based on a ratio between foliage mass and stem sapwood area = 600 kg m^−2^ (Mäkelä 1986) specific leaf area = 4 m^2^ kg^−1^, ratio between total leaf area and crown surface area = 1 (Duursma and Mäkelä 2007), and spherical crown geometry
η_b,2_	−	13		
*m*_1_/*m*_2_/*m*_3_	−	0.02/5/0.7	Constants used in mortality model	Selected to give reasonable width−height relationships
*η*_s_/*η*_b_	kg m^−2^	600/450	Foliage mass to sapwood area ratio	Mäkelä (1986)
*φ*_s_/*φ*_b_	−	0.75/0.7	Form factor	Mäkelä (1986)
*ρ_w_*	kg m^−3^	400	Wood density	Mäkelä (1986)
*θ_cr_*/*θ_fr_*	−	0.2/0.7	Root mass to biomass ratios	Guess/Mäkelä and Valentine (2001)
*θ*_HW,1_	m^−2*θ*HW,2 + 1^	0.0724	Heartwood parameters	Chosen such that when *H*_s_ increases by *H*_B_ all sapwood has been exchanged
*θ*_HW,2_	−	2		
		Light-environment parameters		
*Q*_0_	−	0.23–0.40	Light level at initial tree height	
*dQ*_max_	−	0.071–0.36	Maximum light gradient	
*H*_dQmax_	m	5–30	Height at maximum light gradient	
*a*	−	0.6–0.95	Parameters used in the light environment model	
*b*	m	5–30		
*c*	m^−1^	0.3–2.4		
*d*	−	0.05–0.4		

^*^Index indicate organs: f = foliage, s = stem, b = branches, cr = crown.

### Physiological model of an individual tree

The physiological model used in our simulations is based on the work of Mäkelä (1986) (Eqs (1) and (2)) and considers five biomass pools: stem, branches, foliage, coarse roots and fine roots. The net production $P$ (Eq. (1)) is determined by subtracting from the gross primary production, ${K}_{\mathrm{P}}Q\Big({H}_{\mathrm{tot}}\Big){W}_{\mathrm{f}}$, the maintenance respiration and tissue turnover (adjusted for growth respiration), ${W}_{\mathrm{f}}{K}_{\mathrm{f}}+{W}_{\mathrm{s}}{K}_{\mathrm{s}}+{\mathrm{W}}_{\mathrm{b}}{K}_{\mathrm{b}}+{K}_{\mathrm{cr}}{W}_{\mathrm{cr}}+{K}_{\mathrm{f}\mathrm{r}}{W}_{\mathrm{f}\mathrm{r}}$:

(1) \begin{equation*} P=\frac{1}{1+{r}_G}\left({K}_{\mathrm p}Q\left({H}_{\mathrm tot}\right){W}_{\mathrm f}-{W}_{\mathrm f}{K}_{\mathrm f}-{W}_{\mathrm s}{K}_{\mathrm s}-{W}_{\mathrm b}{K}_{\mathrm b}-{K}_{\mathrm cr}{W}_{\mathrm cr}-{K}_{\mathrm fr}{W}_{\mathrm fr}\right). \end{equation*}

**Figure 2.**
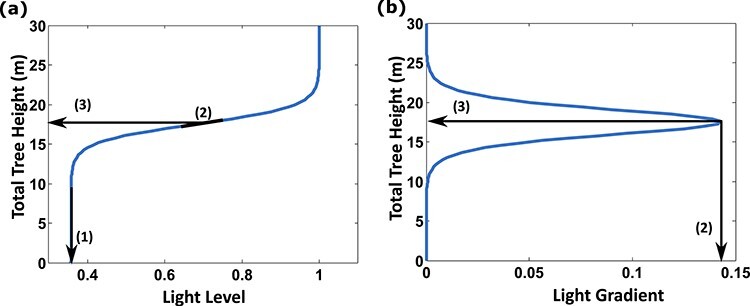
The whole tree is described by the quantities: sapwood area below the main branches *A*_s_, stem height *H*_s_. Heartwood area at ground level *A*_HW_ is determined by the accumulated *A*_s_ and *H*_s_ growth. The tree crown height *H*_b_ is determined by *A*_s_.

Here, *W*_f_, *W*_s_, *W*_b_*, W*_cr_ and *W*_fr_ are the total dry biomass pool for foliage, sapwood in stem, branches, coarse roots and fine roots, respectively; *K*_f_, *K*_s_, *K*_b_  *K*_cr_ and *K*_fr_ are the combined maintenance and turnover costs (adjusted for growth respiration), *r*_G_ is the growth respiration constant (growth respiration, *r*_G_*P*) and *K*_p_ is the maximum gross production rate; parameter values are listed in Table 1. *Q* is the light level relative to that above the canopy at the crown top and *H*_tot_ denotes the total height of the tree. We use the light-use efficiency concept (net production is linearly proportional to absorbed photosynthetically active radiation), which is appropriate for modeling seasonal or longer term productivity (Medlyn 1998). Based on the pipe model (Shinozaki et al. 1964), we assume that the stem sapwood area (*A*_s_) is proportional to the total dry weight of foliage (*W*_f_) and similarly for branch sapwood area. Further, we assume that the ratio of the leaf area to crown surface area, i.e., the surface area of the polygon that encloses the tree crown, and the specific leaf area are constant. With these assumptions gross primary production is proportional to the foliage dry weight, i.e., *P*  $\propto$ crown surface area $\propto$ leaf area $\propto$ leaf biomass. We also assume that the coarse root mass is proportional to stem sapwood biomass and that fine root biomass is proportional to foliage mass (i.e., functional balance), which is reasonable as we do not consider variability in soil resources (Mäkelä 2002). The crown height is assumed to relate to sapwood area (sapwood cross-sectional area) through a power law relation (Mäkelä 1997, Valentine and Mäkelä 2005), ${H}_{\mathrm{b}}={\eta}_{\mathrm{b},2}{A_{\mathrm{s}}}^{\eta_{\mathrm{b},1}}$, where ${\eta}_{\mathrm{b},1}$ and ${\eta}_{\mathrm{b},2}$ are parameters. This means that crown surface area, crown height and sapwood area are linked in a fixed relationship. We motivate this with findings that while stem to crown biomass ratio changes over the course of a tree’s life span, crown shape is relatively stable and crown size remains strongly correlated to stem girth (Harja et al. 2012, Antin et al. 2013), and that Mäkelä and Sievänen (1992) found a power law relation as a result from an optimization study of height growth in open-growth trees. With these assumptions, all the biomass pools can be expressed in terms of two quantities: the crown size and the stem height (*H*_s_; Figure 2). Because the crown size is determined by *A*_s_, we will use *A*_s_ as a proxy for crown size. The relationships between the biomass pools, *A*_s_ and *H*_s_ are described by Eq. 2.

(2) \begin{equation*} {\displaystyle \begin{array}{l}{W}_{\textrm{f}}={\eta}_i{A}_i, where\ i=\textrm{s},\textrm{b},\\{}{W}_i={\varphi}_i{\rho}_\textrm{w}{A}_i{H}_i, where\ i=\textrm{s},\textrm{b},\\{}{W}_{\mathrm{cr}}={\theta}_{\mathrm{cr}}{W}_{\mathrm s},\\{}{W}_{\rm fr}={\theta}_\mathrm{fr}{W}_\mathrm{f}.\end{array}} \end{equation*}

The subscripts in Eq. (2) are abbreviations for branches (b), coarse roots (cr), fine roots (fr) and stem (s). *A*_b_ is defined as sapwood in primary branches. ${\eta}_\mathrm{i},\mathrm{where \ i}=\mathrm{s},\mathrm{b},$ are foliage mass to sapwood area ratios, ${\varphi}_{i},\mathrm{where} \ i=\mathrm{s},\mathrm{b},$ are stem and branch form factors, ${\rho}_{\mathrm{w}}$ is the wood density and ${\theta}_{i},\mathrm{where}\ {i}=\mathrm{cr},\mathrm{fr},$ are root mass to biomass ratios.

### Heartwood dynamics

Aside from the five aforementioned biomass pools we also consider the development of heartwood, defined by heartwood area at the base of the tree trunk (*A*_HW_). The pipe model assumes that every leaf is connected to the roots through a so-called pipe. Because a leaf cannot change its vertical position, when the crown rises, the lower leaves and branches will die off and the associated pipes will disconnect (Shinozaki et al. 1964). We assume that these disconnected pipes constitute the heartwood. Thus, heartwood increases when the stem height increases, i.e., we consider heartwood development to be a growth process as suggested by Bamber (1976). The dynamics of *A*_HW_ are given by differential equation Eq. (3).

(3) \begin{equation*} \frac{dA_{HW}}{dt}={\theta}_{HW,1}{A_s}^{\theta_{HW,2}}\frac{d{H}_s}{dt} . \end{equation*}

In Eq. (3), ${dA}_{\mathrm{HW}}\ \mathrm{and}\ d{H}_{\mathrm{s}}$ are the infinitesimal increments of heartwood and stem height, respectively, and ${\theta}_{\mathrm{HW},1}$ and ${\theta}_{\mathrm{HW},2}$ are power law parameters.

**Figure 3.**
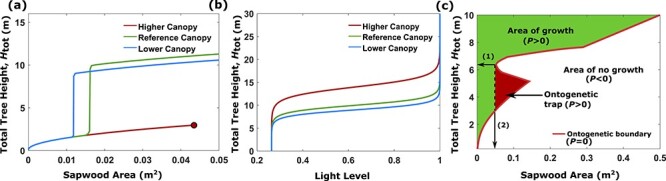
The optimal growth trajectory depends on the whole light environment and a critical threshold in sapwood area determines which trees can reach the canopy top. When the maximum light gradient, *dQ*_max_, is decreased and the height at maximum light gradient, *H*_dQmax_, is increased, the tree will invest more into crown size before switching to stem height and invest more in stem height before switching back to crown size growth. (a) The optimal growth paths and (b) the corresponding light environments for three different cases: reference canopy, lower canopy and higher canopy. For the reference canopy, the height at maximum light gradient is set to 10 m and the maximum light gradient is 0.25 m^−1^. For the lower canopy, the height at maximum light gradient is decreased to 9 m and for the higher canopy, it is increased to 14 m. The corresponding maximum light gradients are 0.27 and 0.17 m^−1^, respectively. The light condition (light level and gradient) at ground level is the same in all three cases. In the left figure a circle signals the end of a growth trajectory and when seed production starts. (c) The ontogenetic plane. The ontogenetic plane is divided into three areas, an area where the tree is able to grow (area of growth), an area where the tree cannot grow (area of no growth) and an area we call the ontogenetic trap (triangular area). Inside the ontogenetic trap which starts at a sapwood area (and associated crown size) indicated by (2), growth is possible, but the tree cannot reach a height larger than the critical tree height (marked (1)), and is unable to reach the canopy.

### Growth dynamics

In our model, allocation is determined by the portions of the net production that is invested in each of stem-height growth and crown size growth (and associated sapwood growth), while the remainder goes to reproduction. We denote by *u*_H_ and *u*_A_ the portion of net production invested in stem height and crown size, respectively, thus *u*_H_, *u*_A_ ≥ 0 and *u*_H_ + *u*_A_ ≤ 1. Importantly, we allow the allocation to the different compartments to change over time and hence both proportions are functions of time, *u*_H_ = *u*_H_(*t*) and *u*_A_ = *u*_A_(*t*), and each such pair of functions corresponds to unique growth trajectory. The part of the net production not invested into stem height or crown size is invested into reproductive output (seed production), i.e., the proportion equal to 1 *u*_H_ *u*_A_. The dynamics of the biomass pools is given by Eq. (4).

(4) \begin{equation*} {\displaystyle \begin{array}{l}\frac{d{W}_i}{dt}=P{u}_i,\\{}{u}_i=\frac{\partial{W}_i}{\partial{H}_s}/\frac{\partial W}{\partial{H}_s}{u}_\mathrm{H}+\frac{\partial{W}_i}{\partial{A}_s}/\frac{\partial W}{\partial{A}_s}{u}_{\mathrm A}, {\mathrm where\ i=\mathrm{f,s,b, cr, fr}}.\end{array}} \end{equation*}

In Eq. (4), ${W}$ is the total dry mass of living tissue, i.e., ${W}={W}_{\mathrm{f}}+{W}_{\mathrm{s}}+{W}_{\mathrm{b}}+{W}_{\mathrm{cr}}+{W}_{\mathrm{fr}}$.

**Figure 4.**
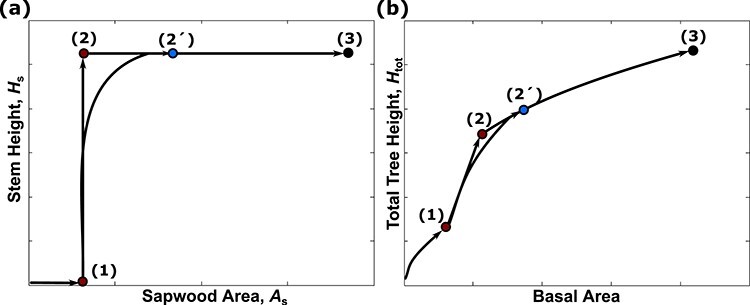
Growth as determined by our heuristic allocation model. (a) The growth in the stem height (*H*_s_)−sapwood area (*A*_s_) plane and (b) the corresponding growth trajectory in the total tree height (*H*_tot_)−basal area plane. Initial allocation is focused on crown size growth. Once the switching crown size (1) is reached, allocation switches to stem height growth. Once the switching stem height is reached (marked (2)), the allocation strategy again switches to crown size. However, if the condition for simultaneous growth is met, the optimal growth trajectory will follow along an arc and allocation gradually shifts from full stem height investment to full crown size investment (marked (2′)). From mark (2)/mark (2′) crown size growth recommences and lasts until an optimal size is reached (marked (3)), where size growth stops, and seed production starts.

### Definition of optimal growth

We assume that the tree allocates biomass to stem height and crown size growth optimally, i.e., in a way that maximizes fitness. Fitness (*J*) is modeled as the lifetime amount of production invested in reproduction, which is equal to the reproductive production at each time t and the probability of survival until that time, integrated over time. Mathematically, this is expressed as:

(5) \begin{equation*} J={\int}_0^{\infty }S(t)P\left(1-{u}_\mathrm{H}(t)-{u}_\mathrm{A}(t)\right)\ dt. \end{equation*}

The probability of the tree still being alive declines over time as:

$$ \frac{dS}{dt}=-m(t)S,$$

where *m*(*t*) denotes the mortality rate. For more information on $S(t)$ see Appendix C available as Supplementary Data at *Tree Physiology* Online.

### Mortality

In our model, we use a size-dependent mortality based on the model by Fridman and Ståhl (2001):

(6) \begin{equation*} m(t)=-\mathit{\ln}\left(1-{m}_1-{m}_2{e}^{-{m}_3\frac{d_0(t)}{d_\mathrm{crit}(t)}}\right). \end{equation*}

In Eq. (6), *m*_1_, *m*_2_ and *m*_3_ are parameters (Table 1) and *d*_0_(*t*) is the stem diameter at ground level, calculated as the diameter of a circle with the area equal to the sum *A*_HW_(*t*) and *A*_S_(*t*). The parameter *d*_crit_(*t*) is the minimum diameter to avoid mechanical failure (Landsberg and Sands 2010), where ${d}_{crit}(t)={\Big(\frac{H_{\mathrm{tot}}(t)}{8.6}\Big)}^{3/2}$and *H*_tot_(*t*) is the height of the tree at time *t*. *m*_1_ represents the base mortality, i.e., the minimum mortality, which is independent of size, and *m*_2_ and *m*_3_ define the size-dependent mortality. While the mortality accounts specifically for mechanical failure, all other mortality risks, such as disease and herbivory, are subsumed in the base mortality.

### Perfect-information approximation

We assume that the tree possesses information on the whole light environment (from the ground to the top of the canopy) when it develops its allocation strategy. That is to say, the function *Q* can be evaluated for the whole light profile to determine the allocation to stem and crown in each time step. We refer to this assumption as the Perfect Information Approximation (PIA).

### Dynamic programming

Dynamic programming is a method used for solving complex decision-making problems, by dividing the main problem into smaller problems and solve these in a recursive manner. A detailed explanation of this method is given in Appendix B available as Supplementary Data at *Tree Physiology* Online. In short, the method discretizes the problem and finds the optimal growth trajectory by trying all possible allocation decisions. In this study, we use dynamic programming for finding the allocation functions *u*_H_(*t*) and *u*_A_(*t*), which maximize the fitness proxy Eq. (5).

### Heuristic allocation method

We propose a heuristic allocation method, based on Pontryagin’s maximum principle (see Appendix C available as Supplementary Data at *Tree Physiology* Online). The method is built on the PIA and consists of two sub-strategies to cope with the two different types of local environmental conditions faced during growth: one for the first phase of growth in shaded or understorey conditions (the shade strategy) and one for growth in high-light conditions just below the canopy (the canopy strategy). The shade strategy begins with crown size growth only up to a switching point (mark 1, Figure 4), where growth switches to stem-height growth only. The next switching point from shade to canopy strategy is triggered when the tree reaches a stem height where a switch to crown size growth (mark 2, Figure 4), or simultaneous investment in crown size and height (mark 2′, Figure 4), becomes optimal (i.e., gradual shift, see case 2 in Appendix C available as Supplementary Data at *Tree Physiology* Online). Growth stops when the tree reaches an optimal final size and seed production starts (mark 3, Figure 4). Based on the PIA, we determine the switching points (mark 1 and 2, in Figure 4) by optimizing them with respect to fitness, i.e., maximizing the fitness proxy (Eq. (5)) using the built-in MATLAB optimization method genetic algorithm.

The difference between dynamic programming and the heuristic method is that for dynamic programming we find the optimal growth trajectory by considering all possible trajectories, or more accurately all possible allocation functions *u*_H_(*t*) and *u*_A_(*t*). In the case of the heuristic method a functional form for the optimal growth trajectory is already given and we merely need to optimize the two strategy switching points.

## Results

### Tree height and diameter growth can be described in terms of three phases

In addition to the numerical optimizations of the growth trajectory, we performed a theoretical analysis of the generality of the findings (Appendix C available as Supplementary Data at *Tree Physiology* Online). The analysis reveals that the sequence of growth phases followed by a reproductive phase, described under Heuristic allocation method above is not only true for our choice of growth model and light function but will hold true for a wider range of growth models (see Appendix C available as Supplementary Data at *Tree Physiology* Online for more details). However, the specific growth trajectory, i.e., the numerical results, will depend on the particular model and parameters applied.

### Heuristic method predicts near-optimal growth trajectories

**Figure 5.**
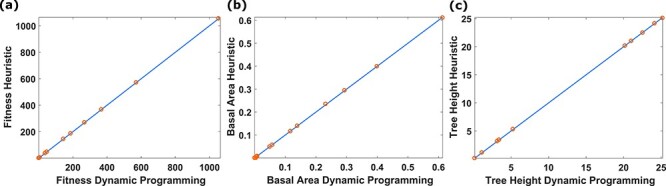
The heuristic model gives good approximation of the optimal growth strategy. We compare the resultants from our heuristic method and dynamic programming for different light environments. Our heuristic method produces results close to dynamic programming, when comparing fitness, the calculated fitness value (a), the tree basal area (b) and the height (c). Each circle represents a unique light environment. The different light environments were generated as described in theory and model.

To test if our heuristic method correctly predicts the optimal trajectory, we compared the growth trajectory predicted by the heuristic method to the trajectories derived by dynamic programming for a wide range of light environments. The close agreement between the results of the two methods indicates that the heuristic method provides a very good approximation of the true optimal growth trajectory (Figure 5).

### The early growth strategy in shade depend on the whole light profile up to the canopy

The first strategic switching point from crown size expansion to stem height growth depends on the whole light profile, in particular, the maximum light gradient and its position (Figure 3a and b). In contrast, a tree that maximizes growth based on only the local light gradient would invest only in crown growth (with no stem height increase) due to the shallow light gradient near the ground.

The height growth strategy (all the switching points) depends on the tree species, i.e., on the physiological parameters. This means that for a given light profile, some species can reach the canopy top according to the strategy described above, while others (with different parameters) cannot. For these species it is therefore optimal to remain at low height, i.e., a sub-canopy strategy (Figure 3a).

### Early crown expansion can lead to an ‘ontogenetic trap’ that prevents the tree from reaching the canopy top

We explored how the canopy light profile, in terms of canopy height and density, affects the optimal growth path (Figure 3a). The steeper the maximum light gradient, and the lower down it occurs, the smaller is the crown size at which the tree switches to height growth and the lower the stem height at which crown expansion resumes again toward the top of the canopy. When the maximum light gradient (steepest increase in light) is too high up, the optimal growth path is to remain at ground level and only invest into crown size growth. A critical threshold in sapwood area determines which trees can reach the top of the canopy, and to illustrate this, we define an ontogenetic plane with sapwood area on the horizontal axis and total tree height on the vertical axis. Calculating the net production at every point, we found that the plane is divided into two regions. The first region is where the net production is negative ($P<0$), and the tree cannot sustain its body size, and consequently must reduce its size. The second region is where the net production is positive ($P>0$), and the tree will grow. On the border between these two regions the net production is zero ($P=0$) (Figure 3c). The size of these regions depends on the physiological model parameters, i.e., the size differs among tree species. Trees can only grow within the positive growth region with increasing height and increasing or constant sapwood area, toward a point near or at the top of the canopy where they stop growing and start reproductive production. However, they can also get stuck on a much lower height due to an ontogenetic trap created by the shape of the zero-growth border (Figure 3c). The trap is associated with a threshold crown size (marked (2) in Figure 3c); any growth trajectory crossing this threshold will be unable to grow taller than a critical tree height (marked (1) in Figure 3c).

The ontogenetic ‘trap’ is best explained by the relationship between light gradient (the light increase with tree height) and the associated photosynthetic gain. Because of the shape of the light profile, the change in light level is shallow at low tree height, leading to a small gain in gross production with height growth. The maintenance cost, however, always increases linearly with tree height and may exceed the photosynthetic gain and lead to a decrease in net production with height. This trend will continue as long as the light gradient is below the maintenance cost increment, i.e., until the light gradient gets steeper higher up toward the canopy. An optimal growth path must avoid growing into this trap, in order to reach canopy height.

The ontogenetic trap will become larger when the maximum light gradient is reduced, or is positioned higher up, i.e., when growing under taller trees with a higher canopy. If the maximum light gradient is very small, or it is positioned too high up, the ontogenetic trap will cut through the ontogenetic plane and divide it into two regions, making it impossible for the tree to reach the canopy, which means that the optimal strategy is not to grow up to the canopy.

### The model predicts taller trees and increased investment into crown size when wood density decreases

**Figure 6.**
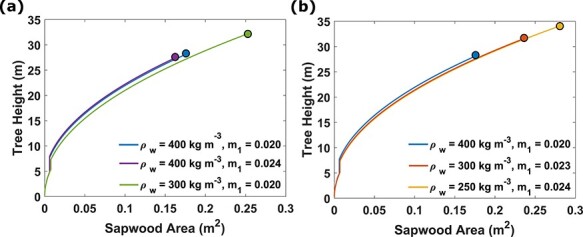
The model predicts that a tree with lower wood density (*ρ*_w_) and higher mortality (*m*_1_) grows to a larger size. We compare growth trajectories for trees with lower wood density or higher mortality (a) and examine the change in growth trajectory when wood density and mortality are changed simultaneously (b). The relationship between the wood change in density and mortality was calculated $\Delta{m}_1=-2.55\times{10}^{-5}\Delta{\rho}_{\mathrm w}$, and the number $-2.55\times{10}^{-5}$ m^3^ kg^−1^ was determined as the mean slope from Figure 1 in Iida et al. (2012). The circle at the end of each growth trajectory signals the final size, and when seed production starts. All trees were subjected to the same light condition (height at maximum light gradient, *H*_dQmax_ = 10 m, maximum light gradient, *dQ*_max_ = 0.25 m^−1^ and initial light level, *Q*_0_ = 0.27).

Beside the light environment, the optimal growth path is determined by the values of the parameters in the physiological growth model. We assess the effect of wood density and mortality (*m*_1_) on the switching points (Figure 6). Decreasing wood density resulted in increased initial crown expansion before switching to height growth, earlier switching to final crown expansion in the canopy and increased final tree size (Figure 6a). We found that increasing mortality had a small effect on the above switching points, but there was a noticeable reduction of the final tree size (Figure 6a). When the wood density is decreased and mortality increased simultaneously according to a relationship observed across species (Iida et al. 2012), the effects are similar to the effect of only reducing wood density (Figure 6b).

## Discussion and conclusion

### Simple heuristic strategies predict optimal growth to the top of the canopy

Growing the crown size beyond a certain critical threshold at low height will render the tree unable to reach the canopy, i.e., it will be trapped at a sub-canopy height. This critical threshold is defined by a bottleneck in terms of a growth rate minimum in the ontogenetic plane defined by stem height and crown size. These findings imply that existing optimization approaches based on local information (Perrin 1992, Dewar et al. 2009, Franklin et al. 2012) are not sufficient to predict variation in stem and crown diameter in individual-based forest models. Instead, the two-stage optimization model presented here provides a solution. Alternatively, dynamic programming could be used, which, however, suffers from heavy computation load and does not provide insights into the determinants of the optimal strategy. In comparison with the most commonly used allocation model—static allometric relationships—our method has two decisive advantages: the optimization eliminates the need for species-specific allometric parameters, and it automatically adjusts allocation behavior to changing environmental conditions.

### Optimal height growth explains key aspects of tree-height distributions in forests

The tree growth literature comprises countless studies of tree growth in even-aged stands (in full light) (Bergh et al. 1999, Binkley et al. 2010), some short-term studies of seedlings in controlled light conditions (Kubiske and Pregitzer 1996, Poorter et al. 2005), and a few long-term studies in forests under unknown light conditions (Pretzsch 2005). Remarkably, however, we did not find any published observations of growth paths in terms of height and crown size (or stem diameter) for trees grown toward increasing light under measured light conditions.

From the simulations we observe that if the canopy density is below, or the canopy height is above, a certain critical threshold then it will no longer be profitable (in terms of fitness) to grow up to canopy height, but rather to develop only crown size, creating an understory layer in the forest, see Figure 3. The critical threshold is sensitive to the physiological parameters, which vary with species. Thus, the threshold varies among species, which implies that in a given light environment some species may follow a sub-canopy strategy, whereas others should strive to reach the canopy, resulting in height-stratified forests as often observed (e.g., Cermák 1998, Batista et al. 2014). The model also offers an explanation for why canopy species do not grow crowns as large as sub-canopy species of the same height (e.g., Bohlman and O’Brien 2006), and why crown size stops growing if their height growth has stalled in the shade (Kubota et al. 1994)—increased crown size would impair continued height growth when light conditions improve.

In contrast to some other studies of height growth strategies (e.g., Mäkelä 1985, King 1990, Falster and Westoby 2003), we do not account for the feedback of the trees’ strategy on their own light environment, which explains the evolution of stems and light competition in forests as an evolutionarily stable strategy (ESS). This, however, does not mean that our model is inconsistent with ESS models, instead it addresses a different question: although ESS models usually converge to an equilibrium strategy and an associated generated light environment, in reality a tree species or genotype will face unpredictable and variable environments. Our model predicts how a tree responds by plasticity to such variation, although it does not explain what the tree’s evolved mean ESS environment is. However, although the model does not derive the full forest ESS environment, it provides an explanation why sub-canopy species have evolved in the presence of taller canopy species due to their diverging height versus crown size growth strategies.

### Model predicts similar trend between wood density and tree diameter as observed in empirical data

When mortality was increased and wood density remained constant, the switching points remained largely unchanged. However, the switch from growth to reproduction occurred earlier, which results in a decrease in the final tree size. This trend is reasonable as increasing mortality (*m*_1_) will decrease the probability of survival, which has a negative effect on fitness (see Appendix D available as Supplementary Data at *Tree Physiology* Online).

When the wood density decreased, and mortality remained constant, we observed an increase in sapwood investment and an increase in the final tree size. Looking at the model we see that decreased wood density will lead to an increase in net production, which in return will increase growth rate. A decrease in wood density will also cause an increase in the region of positive net productivity (see Figure 3c) and the point in the ontogenetic plane of maximum net productivity will appear at a larger crown size. Thus, it is reasonable that a decrease in wood density would result in an increase in crown size as this would increase fitness (see Appendix D available as Supplementary Data at *Tree Physiology* Online).

A number of empirical studies have observed a negative correlation between mortality and wood density (Poorter 2008, Kraft et al. 2010) and if we are using a linear relation between wood density and base mortality (*m*_1_), the model predicts an increased investment into crown size, both at an early stage before stem height growth starts and later, during the final crown expansion in the canopy. We also observe that at a given tree height, a decrease in wood density leads to an increase in the total sapwood area and stem diameter. This is consistent with empirical observations made by Iida et al. (2012), who found a negative correlation between wood density and stem diameter when different tree species are compared. However, the study also observed a positive correlation between wood density and crown width, which is not the case in our model because we assume that the crown geometry is the same among all species.

### How much can a tree know—the PIA

Regarding a tree’s ability to sense its environment, one can imagine a range of different levels, with two hypotheses serving as the extremes. As one extreme, we can assume that the tree possesses no knowledge about its environment. This has the consequence that no matter the environment, the tree morphology will always follow a genetically preprogrammed allometric trajectory. The other extreme is our PIA assumption, that the tree possesses absolute knowledge of its surroundings (the light environment in our case). This assumption allows the tree to change morphology and be plastic in response to environmental variation. Out of the two extreme hypotheses it could be argued that the latter is closer to reality (e.g., Trewavas 2005, Gagliano et al. 2014). This would also be expected given the advantages shown here of being able to sense of the full light environment, which should result in a strong selection pressure for this trait.

### Outlook

The key benefit of our allocation model, compared with traditional allocation methods such as static allometric functions, lies in its ability to explain and predict changes of growth allocation in response to variations in the surrounding environment. This aspect is important for a variety of fields, from natural vegetation modeling to forestry. For example, in forestry applications, it is of great importance to realistically simulate the impact of changes in the light environment on productivity and diameter growth in response to planting density or after a thinning operation. Thus, the model can provide a vital component for individual-based forest models, and model-aided decision tools for optimizing thinning in forestry planning.

In our current model we do not consider any plasticity in the crown shape and we do not consider the light variation within the tree’s crown. In a more realistic setting, one would include crown area as an independent variable to account for crown plasticity where the tree can optimize the leaf area directly exposed to sunlight and self-shading inside of the crown architecture. However, the model can readily be extended, e.g., to include biomass investment in additional organs or optimal adjustment of additional traits (see Appendix C available as Supplementary Data at *Tree Physiology* Online). In our current model we focused on two traits, stem height and sapwood area, and a natural extension would be to include crown height and crown width.

While our model describes growth strategies in newly formed gaps, it does not apply to growth under gap formation, i.e., it does not account for dynamic competition between the focal tree and its neighbors. However, although it is beyond the scope of this paper, in principle our model can be applied to gaps if the optimal strategy is re-evaluated in each time step according to the increasing canopy height. On the other hand, some prominent forest models assume that canopy gaps are always filled by growth and stem bending of surrounding trees, i.e., the perfect plasticity approximation (e.g., Purves et al. 2008, Strigul et al. 2008). Such a representation of forest structures could therefore readily be combined with our model of optimal height and diameter growth.

Our model can also be of use to complement other general forest growth models (e.g., Pacala et al. 1996, Falster et al. 2011) to provide further insight into the role of height and crown plasticity in the development and structure of forests. However, the importance of height growth strategy in response to light environment is in stark contrast to the lack of relevant observations, which suggests an urgent need for more observational studies of tree growth patterns under variable light conditions.

## Supplementary Material

Appendix_rev_clean_tpaa110
