# Supplementary material for: A tree’s quest for light—optimal height and diameter growth under a shading canopy
Source: Tree Physiol. 2020 Sep 2;41(1):1–11. doi: 10.1093/treephys/tpaa110 (PMC7868666; doi:10.1093/treephys/tpaa110)
Supplement: Appendix_rev_clean_tpaa110 [file appendix_rev_clean_tpaa110.docx]

## Supplementary material

## Appendix A

Light profile

The light profile relative to the top of the canopy is defined by equation A. 1.

|  | $Q\left( H_{tot} \right)=\frac{a}{1+e^{-c\left( H_{tot}-b \right)}}+d.$ | A. 1 |
| --- | --- | --- |

Here *a*, *b*, *c*, and *d* are parameters, which uniquely define the light environment in terms of three properties:, the light level *Q*_0_ at initial tree height, *H*_0_, the maximum light gradient *dQ*_max_, and the height at maximum light gradient, *H*_dQmax_ (fig. 2). The relationship between the parameters and the four properties are given by A. 2.

|  | $\begin{matrix} a+d=1, \\ H_{dQmax}=b, \\ {dQ}_{max}=\frac{ac}{4}, \\ Q_{0}= \frac{a}{1+e^{-c(H_{0}-b)}}+d. \end{matrix}$ | A. 2 |
| --- | --- | --- |

The values of the four parameters are uniquely defined by solving this system of non-linear equations.

## Appendix B

Dynamic Programming

We followed the algorithm described by Kirk (2012) to solve our optimization problem. In this scheme, time, state (in this case the state-variables are sapwood area *A*_s_, the stem height *H*_s_, and the heart wood area *A*_HW_), and control-space (the variables *u*_H_ and *u*_A_) are discretized. We let Δt be the time step of the discrete temporal axis, and ***u***_H_ and ***u***_A_ are finite sets including all possible discrete controls. In order to deal with the fact that our time interval is infinite (in
Eq. 5 we integrate from 0 to ∞), we choose a stop time *T* that is chosen sufficiently ahead of the start time as to have only negligible influence on the outcome. Let *N* be the number of time steps and let $t_{0}=0, t_{i}=i\Delta t, i=\left\{ 1,2,\cdots,N-1 \right\}{,t}_{N}=T$. During the time interval $[t_{i},t_{i}+\Delta t), i= \left\{ 0,\ldots,N \right\}$ we assume that the allocation is constant, thus the fitness proxy, Eq. 5, is reduced to the discrete form:

$$V\left( 0 \right)=\sum_{i=0}^{N} S\left( t_{i} \right)b\left( t_{i} \right)\Delta t.$$

Following the principle of optimality (Bellman 1954) the problem of maximizing *V*(0) can be transformed into recursive form. The recursive form is given by:

$$V\left( t_{i} \right)=\max_{u_{H}\in\boldsymbol{u}_{H},u_{A}\in\boldsymbol{u}_{A}} \left( S\left( t_{i} \right)b\left( t_{i} \right)\Delta t+V\left( t_{i}+\Delta t \right) \right),$$

with the terminal condition *V*(*t_N_*)=0 for all feasible states (every possible combination of stem height and sapwood area). The recursive form is derived from the fact that from any time $t_{i}$ the following allocation decisions needs to be optimal, in order for the whole life-cycle to be optimal. The algorithm for solving this optimization problem, i.e. finding the sequence *u*_H_ and *u*_A_ that maximizes $V\left( 0 \right)$, comprises of the following steps:

**Step 1**. Set the current time, *t*_current_, to *t*_current_ = *T*−Δ*t*. For every feasible state try all possible control decisions and calculate V(*t*_current_), remember *V*(*t_N_*)=0. Save the maximum value of *V*(*t*_current_) and the control decision associated with this value for every state.

**Step 2.** Set previous time, *t*_old_, to *t*_old_ = *t*_current_ and update current time, *t*_current_ = *t*_old_−Δ*t*. Again, for every feasible state try all possible control decisions and calculate *V*(*t*_current_), use the previous calculated values of *V*(*t*_old_). Save the maximum value of *V*(*t*_current_) and the control decision associated with this value, for every state. Repeat this step until *t*_current_ = *t*_0_.

The result from this algorithm is a look-up table containing the optimal control decision for every possible state at any discrete time step *t*. An optimal growth trajectory can thus be constructed by applying the information from the look-up table at the initial state and sequentially build the growth trajectory from the table from *t_0_* to *t_N_*.

## Appendix C

Deriving necessary conditions for optimal growth, for a general growth model

Below follows an analysis of a general growth model to derive necessary conditions for optimal growth. We start by introducing notations and continue with introducing a general growth model. For the general model we consider the tree to be defined by a number of quantities, e.g. stem height and crown size. Next, we define the optimization problem, i.e. how biomass should be optimally distributed among the different quantities. Lastly we derive necessary conditions for optimal allocation by applying Pontryagin’s maximum principle (Pontryagin 1962; Halkin 1974; Aseev and Kryazhimskii 2003; Kirk 2004). In contrast to dynamic programming, which solves the allocation problem but doesn’t give insight to the optimal strategy, Pontryagin’s maximum principle gives us necessary conditions for the optimal trajectory. However, these conditions might not be sufficient to describe the optimal allocation.

The general growth model

For the general model we assume a tree be defined by $N\mathbb{\in N}$ independent quantities (e.g. stem height, crown height, crown width, root biomass pool, etc.) we denote these as $x_{i}, where i\in\left\{ 1,2,\ldots,N \right\}$. We assume the total biomass of the tree is given by a function $W(x_{1},\cdots,x_{N})$, the net production by a function $P(x_{1},\cdots,x_{N})$, and that a fraction $\tilde{u}$ of the net production is invested into growth. We also introduce the useful relationship:

|  | $\frac{\alpha_{j}}{\alpha_{i}}\frac{\partial\alpha_{i}}{\partial x_{j}}=\frac{\alpha_{i}}{\alpha_{j}}\frac{\partial\alpha_{j}}{\partial x_{i}} i,j\in\left\{ 1,2,\ldots,N \right\}.$ | A. 3 |
| --- | --- | --- |

Here $\alpha_{i}$ is $\left( \frac{\partial W}{\partial x_{i}} \right)^{-1}$. The time derivative of $W$ is given by:

$$\frac{dW}{dt}=P\tilde{u}=\sum_{i=1}^{N} \frac{\partial W}{\partial x_{i}}\frac{dx_{i}}{dt}, 0\leq\tilde{u}\leq1.$$

If $u_{i}$is the fraction of the net production invested into $x_{i}$, and

$$\tilde{u}=\sum_{i=1}^{N} u_{i},$$

then we get the following equation

|  | $P\sum_{i=1}^{N} u_{i}=\sum_{i=1}^{N} \frac{\partial W}{\partial x_{i}}\frac{dx_{i}}{dt}.$ | A. 4 |
| --- | --- | --- |

Because every $u_{i}$ only effects the corresponding $x_{i}$, we can equate each term in sum on the left-hand side of A. 4 to the corresponding term on the right-hand side and we get the dynamics for $x_{i}$ as:

$$\frac{\partial W}{\partial x_{i}}\frac{dx_{i}}{dt}=Pu_{i}\Leftrightarrow\frac{dx_{i}}{dt}=\alpha_{i}Pu_{i}.$$

The allocation problem and Pontryagin’s maximum principle

The optimization problem is to find the piecewise continuous functions $u_{i}(t)$, i.e. the allocation schedule, that maximizes the performance measure (fitness proxy):

$$J\left( u_{1}\left( t \right),\cdots,u_{N}(t) \right)=\int_{0}^{\infty} S\left( t \right)P\left( x_{1}\left( t \right),\cdots,x_{N}\left( t \right) \right)\left( 1-\sum_{i=1}^{N} u_{i}\left( t \right) \right) dt.$$

Here, *S* is a function representing the probability of surviving to time *t* and the dynamics of *S* is given by:

$$\frac{dS}{dt}=-mS, S_{0}=1.$$

Here, $m$ denotes the mortality rate and is assumed to be a function $m(x_{1},\cdots,x_{N})$, and thus changes with time. We can write the solution of the equation above as:

$$S=S_{0}e^{-\int_{0}^{t} m\left( x_{1}\left( s \right),\cdots,x_{N}\left( s \right) \right) ds}.$$

By definition (Kirk 2004) the Hamiltonian for our model is given by:

$$\mathcal{H}\left( u_{1}\left( t \right),\cdots,u_{N}(t),x_{1}\left( t \right),\cdots,x_{N}\left( t \right),S\left( t \right),\lambda_{s}\left( t \right),\lambda_{1}\left( t \right), \cdots,\lambda_{N}\left( t \right) \right)=S\left( t \right)P\left( x_{1}\left( t \right),\cdots,x_{N}\left( t \right) \right)\left( 1-\sum_{i=1}^{N} u_{i}\left( t \right) \right)+\sum_{i=1}^{N} \lambda_{i}\frac{dx_{i}}{dt}-\lambda_{S}\left( t \right)mS\left( t \right).$$

If ${u_{1}}^{*}\left( t \right),\cdots,{u_{N}}^{*}(t)$ are the control trajectory maximizing $J$ and ${x_{1}}^{*}\left( t \right),\cdots,{x_{N}}^{*}\left( t \right)$ are the corresponding optimal growth trajectory then Pontryagin’s maximum principle states that there exist functions $\lambda_{S}^{*}$ and $\lambda_{1}^{*}, \cdots,\lambda_{N}^{*}$ satisfying the following conditions (Halkin 1974):

$$\begin{matrix} \frac{d\lambda_{i}^{*}}{dt}=-\frac{\partial\mathcal{H}}{\partial{x_{i}}^{*}},i\in\left\{ 1,2,\ldots,N \right\}, \\ \frac{d\lambda_{S}^{*}}{dt}=-\frac{\partial\mathcal{H}}{\partial S}=\lambda_{S}^{*}m\left( {x_{1}}^{*}\left( t \right),\cdots,{x_{N}}^{*}\left( t \right) \right)-P\left( {x_{1}}^{*}\left( t \right),\cdots,{x_{N}}^{*}\left( t \right) \right)\left( 1-\sum_{i=1}^{N} {u_{i}}^{*}\left( t \right) \right), \\ \mathcal{H}\left( {u_{1}}^{*},\cdots,{u_{N}}^{*},{x_{1}}^{*},\cdots,{x_{N}}^{*},S^{*},\lambda_{s}^{*},\lambda_{1}^{*}, \cdots,\lambda_{N}^{*},t \right)\mathcal{\geq H}\left( u_{1},\cdots,u_{N},{x_{1}}^{*},\cdots,{x_{N}}^{*},S^{*},\lambda_{s}^{*},\lambda_{1}^{*}, \cdots,\lambda_{N}^{*},t \right). \end{matrix}$$

$\lambda_{S}^{*}$ and $\lambda_{1}^{*}, \cdots,\lambda_{N}^{*}$ are also known as the costate variables and their corresponding time derivatives are known as the costate equations. The costate variables ($\lambda_{i}$) indicate sensitivity of fitness ($J$) to a small change in the corresponding quantity ($x_{i}$) (Perrin and Sibly 1993).

For the remaining part of this section we will omit the star-* notation for readability.

To find the controls that maximize $\mathcal{H}$, we start by differentiating the Hamiltonian with respect to the controls *u_i_*:

$$\frac{\partial\mathcal{H}}{\partial u_{i}}=\left( \alpha_{i}\lambda_{i}-S \right)P.$$

The partial derivatives of the Hamiltonian, with respect to each $u_{i}$, do not depend on the controls themselves, in other words the Hamiltonian is linear with respect to the controls. This means the optimal controls are only dependent on the relationship between the $\alpha_{i}\lambda_{i}$’s and $S$. If $a_{i}\lambda_{i}>S$ for at least one *i* then the Hamiltonian is maximized by $\sum_{i=1}^{N} u_{i}\left( t \right)=1$ and we allocate all resources to the quantity with the highest value of $\partial\mathcal{H/}\partial u_{i}$. On the other hand, if $\alpha_{i}\lambda_{i}<S$, for all $i=1,\cdots N$, then the control $\sum_{i=1}^{N} u_{i}\left( t \right)=0$ maximizes the Hamiltonian and all resources are invested into reproductive output (seed production). In short as long as $\alpha_{i}\lambda_{i}>S$ for some *i* then the tree will invest all resources into growth and when $a_{i}\lambda_{i}<S$ growth stops, and reproduction starts. We will study three different cases to cover different relationships between the $\alpha_{i}\lambda_{i}$’s and $S$:

Case 1

In the first case we assume:

$$\alpha_{i}\lambda_{i}>\alpha_{i}\lambda_{j}, for all j\neq i$$

$$\alpha_{i}\lambda_{i}>S.$$

The optimal steering during this period is achieved by the controls $u_{i}=1$ and $u_{j}=0$. We can then write the Hamiltonian as:

$$\mathcal{H=}\lambda_{i}\alpha_{i}P-\lambda_{S}mS.$$

During this period the time derivative of $\lambda_{S}$ is:

$$\frac{d\lambda_{S}}{dt}=\lambda_{S}m.$$

This ODE is satisfied by $\lambda_{S}=1/S$; Thus, the Hamiltonian can be further simplified to:

|  | $\mathcal{H=}\lambda_{i}\frac{dx_{i}}{dt}-m=\lambda_{i}\alpha_{i}P-m.$ |  |
| --- | --- | --- |

The time derivative of the costate variable is given by:

$$\frac{d\lambda_{i}}{dt}=\frac{\partial m}{\partial x_{i}}-\lambda_{i}\frac{\partial\left( \alpha_{i}P \right)}{\partial x_{i}}.$$

This differential equation is satisfied by $\lambda_{i}=m/$($\alpha_{i}P$). We verify this by inspecting the time derivative of $m/$($\alpha_{i}P$).

$$\begin{matrix} \frac{dm/(\alpha_{i}P)}{dt}=\dot{m}\frac{1}{\alpha_{i}P}-\frac{m}{\left( \alpha_{i}P \right)^{2}}\dot{\left( \alpha_{i}P \right)}=\sum_{j=1}^{N} \left( \frac{\partial m}{\partial x_{j}}\frac{dx_{j}}{dt} \right)\frac{1}{\alpha_{i}P}-\frac{m}{\left( \alpha_{i}P \right)^{2}}\sum_{j=1}^{N} \left( \frac{\partial\left( \alpha_{j}P \right)}{\partial x_{j}}\frac{dx_{j}}{dt} \right)= \\ =\frac{\partial m}{\partial x_{i}}\alpha_{i}P\frac{1}{\alpha_{i}P}-\frac{m}{\left( \alpha_{i}P \right)^{2}}\frac{\partial\left( \alpha_{i}P \right)}{\partial x_{i}}\alpha_{i}P \end{matrix}$$

This satisfies the conditions of the maximum principle.

Case 2

For the second case we introduce a subset of indices $I\subseteq\left\{ 1,2,\ldots,N \right\}$ and consider the condition:

|  | $\alpha_{i}\lambda_{i}=\alpha_{j}\lambda_{j} for all i,j\in I$ | A. 4 |
| --- | --- | --- |

and

$$\alpha_{i}\lambda_{i}>\alpha_{j}\lambda_{j} for all i\in I, j\notin I$$

$$\alpha_{i}\lambda_{i}>S$$

If the subset $I$ includes more than one element and the condition given above, A. 4, is only valid for a single point in time, then case 2 corresponds to an instantaneous switching point in allocation priority, e.g. the shift from stem-height investment to crown-size investment. If, however, the conditions can last for a finite time period then we have simultaneous investment to multiple quantities; this period is known as a singular arc. To make the condition last over a finite time-period we have to impose a second condition, namely:

|  | $\frac{{d(\alpha}_{i}\lambda_{i})}{dt}=\frac{{d(\alpha}_{j}\lambda_{j})}{dt},i,j\in I.$ | A. 5 |
| --- | --- | --- |

For this case the Hamiltonian is maximized by $u_{i}\left( t \right)\geq0$ for all $i\in I$ and $u_{j}\left( t \right)=0$ for all $j\notin I$. Under these conditions the Hamiltonian becomes:

$$\mathcal{H=}\sum_{i\in I} \lambda_{i}\frac{dx_{i}}{dt}-\lambda_{S}mS.$$

If we calculate the time derivative of $\lambda_{S}$, we get:

$$\frac{d\lambda_{S}}{dt}=\lambda_{S}m.$$

This ODE is satisfied by $\lambda_{S}=1/S$; thus, the Hamiltonian can be further simplified to:

$$\mathcal{H=}\sum_{i\in I} \lambda_{i}\frac{dx_{i}}{dt}-m.$$

By Pontryagin’s maximum principle the costate equations can be expressed by:

$$\frac{d\lambda_{i}}{dt}=\frac{dm}{dx_{i}}-\sum_{j\in I} \lambda_{j}\frac{\partial\left( dx_{j}/dt \right)}{\partial x_{i}}=\frac{dm}{dx_{i}}{-\lambda}_{i}\alpha_{i}\frac{\partial P}{\partial x_{i}}-\sum_{j\in I} \lambda_{j}\frac{\partial\alpha_{j}}{\partial x_{i}}Pu_{j}.$$

If we use the fact that

$$\frac{d\alpha_{i}}{dt}=\sum_{j\in I} \frac{\partial\alpha_{i}}{\partial x_{j}}\frac{dx_{j}}{dt}=\sum_{j\in I} \frac{\partial\alpha_{i}}{\partial x_{j}}\alpha_{j}Pu_{j},$$

, A.3, and A. 4, we can write the time derivative of $\alpha_{i}\lambda_{i}$ as

$$\frac{{d(\alpha}_{i}\lambda_{i})}{dt}=\alpha_{i}\left[ \frac{dm}{dx_{i}}-\lambda_{i}\alpha_{i}\frac{\partial P}{\partial x_{i}}-\lambda_{i}\sum_{j\in I} \frac{\alpha_{i}}{\alpha_{j}}\frac{\partial\alpha_{j}}{\partial x_{i}}Pu_{j} \right]+\lambda_{i}\sum_{j\in I} \frac{\alpha_{i}^{2}}{\alpha_{j}^{2}}\frac{\partial\alpha_{j}}{\partial x_{i}}\alpha_{j}Pu_{j}.$$

If we once more apply the first condition (A. 4) and the relation A. 3, we finally get:

$$\frac{{d(\alpha}_{i}\lambda_{i})}{dt}=\alpha_{i}\left( \frac{dm}{dx_{i}}-\lambda_{i}\alpha_{i}\frac{\partial P}{\partial x_{i}} \right).$$

The final condition (A. 5), gives the relation:

$$\alpha_{i}\left( \frac{dm}{dx_{i}}-\lambda_{i}\alpha_{i}\frac{\partial P}{\partial x_{i}} \right)=\alpha_{j}\left( \frac{dm}{dx_{j}}-\lambda_{j}\alpha_{j}\frac{\partial P}{\partial x_{j}} \right).$$

Using this relation, we can write the costate equations as:

$$\frac{d\lambda_{i}}{dt}=\sum_{j\in I} \frac{\alpha_{j}}{\alpha_{i}}\left( \frac{dm}{dx_{j}}-\lambda_{j}\alpha_{j}\frac{\partial P}{\partial x_{j}} \right)u_{j}-\lambda_{j}\frac{\partial\alpha_{j}}{\partial x_{i}}Pu_{j}.$$

As in case 1 these differential equations are satisfied by $\lambda_{i}=m/(P\alpha_{i})$. We verify this by inspecting the time derivative of $m/$($\alpha_{i}P$):

$$\begin{matrix} \frac{d\left[ m/(P\alpha_{i}) \right]}{dt}=\frac{1}{P\alpha_{i}}\dot{m}-\frac{m}{\left( P\alpha_{i} \right)^{2}}\dot{\left( P\alpha_{i} \right)}= \\ =\frac{1}{P\alpha_{i}}\sum_{j\in I} \frac{dm}{dx_{j}}\alpha_{j}Pu_{j}-\frac{m}{\left( P\alpha_{i} \right)^{2}}\sum_{j\in I} \left( \frac{\partial\alpha_{i}}{\partial x_{j}}P+\alpha_{i}\frac{\partial P}{\partial x_{j}} \right)\alpha_{j}Pu_{j}= \\ =\sum_{j\in I} \frac{\alpha_{j}}{\alpha_{i}}\left( \frac{dm}{dx_{j}}-\frac{m}{P}\frac{\partial P}{\partial x_{j}} \right)u_{j}-\frac{m}{P\alpha_{j}}\frac{\partial\alpha_{j}}{\partial x_{i}}{Pu}_{j}. \end{matrix}$$

This satisfies the conditions of the maximum principle.

Finally, we get the condition for the singular arc:

$$\alpha_{i}\left( \frac{dm}{dx_{i}}-\frac{m}{P}\frac{\partial P}{\partial x_{i}} \right)=\alpha_{j}\left( \frac{dm}{dx_{j}}-\frac{m}{P}\frac{\partial P}{\partial x_{j}} \right).$$

Or equivalent, by multiplying both sided by *–P/m^2^*:

|  | $\alpha_{i}\frac{\partial\left( P/m \right)}{\partial x_{i}}=\alpha_{j}\frac{\partial\left( P/m \right)}{\partial x_{j}}{\Leftrightarrow\left. \frac{d\left( P/m \right)}{dW} \right\vert}_{x_{j\neq i}}=\left. \frac{d\left( P/m \right)}{dW} \right\vert_{x_{i\neq j}}.$ | A. 6 |
| --- | --- | --- |

In equation A. 6 $\left. \frac{d\left( P/m \right)}{\mathrm{dW}} \right|_{x_{j\neq i}}$is the derivative of $P/m$ with respect to $W$ while holding all quantities except $x_{i}$ constant. The equation states that for simulations investment to exist, the change in net production to mortality ratio ($P/m$) with respect to total biomass ($W$) must be equal for two or more quantities ($x_{i}$), i.e. when the investment (in terms of biomass) into two or more quantities have the same effect on $P/m$. On the singular arc the controls have to be chosen such that this equality, A. 6, holds.

Case 3

For the final case we assume that:

$$\alpha_{i}\lambda_{i}<S i\in\left\{ 1,2,\ldots,N \right\}.$$

The optimal control during this period is given by $\sum_{i=1}^{N} u_{i}\left( t \right)=0$. We write the Hamiltonian as:

$$\mathcal{H=}SP_{TR}-\lambda_{S}m_{TR}S.$$

*TR* denotes the time when the growth stopped. $P_{TR}$ and $m_{TR}$ represents the net production and the mortality when growth stopped, these values will remain constant while $\sum_{i=1}^{N} u_{i}\left( t \right)=0$. The time derivative of $\lambda_{S}$ is given as:

$$\frac{d\lambda_{S}}{dt}={\lambda_{S}m_{TR}-P}_{TR}.$$

Which is satisfied by $\lambda_{S}=P_{TR}/m_{TR}$. This satisfies the conditions of the maximum principle.

Summary

To summarize the main findings from the analysis, we find the optimal growth trajectory exhibits the following characteristics 1) Growth and reproduction are separated -the tree life-cycle is divided into a growth period, where all resources are allocated towards growth, and a reproductive period where all investment is channeled towards seed production. 2) Simultaneous investment into two or more quantities is triggered when investment to the quantities in question have equal effects on the production to mortality ratio (*P*/*m*), and if the allocation can be carried out such that these effects remain equal, see equation A. 6. If these two conditions are not satisfied the tree invests all resources into one quantity. The period of simultaneous growth can be represented as a curved trajectory in the ontogenetic plane. These properties are not only true for the growth model presented in the Theory and model section, and/or the light function chosen by us, but for any growth model that can be expressed in the form of the general model.

## Appendix D

Consequences of the optimal controls on the fitness proxy

In Appendix C we concluded that the growth and reproductive phase are divided, meaning at a time $t=t_{R}$ the tree switches from growth to reproduction. When $t<t_{R}$ the controls $u_{H}+u_{A}=1$ and $u_{H}+u_{A}=0$ When $t\geq t_{R}$, this implies that the fitness proxy can be simplified as

$$\int_{0}^{\infty} P(t)S\left( t \right)\left( 1-u_{H}+u_{A} \right)dt=P_{t_{R}}S_{t_{R}}\frac{e^{-m_{t_{R}}t_{R}}}{m_{t_{R}}},$$

here $P_{t_{R}}$ and $m_{t_{R}}$ are the net production and mortality rate at $t_{R}$, and

$$S_{t_{R}}=\int_{0}^{t_{R}} S\left( t \right)dt$$

is the survival probability at $t_{R}$. There are some important things to note: 1) $P_{t_{R}}$, and $m_{t_{R}}$ are independent of the whole growth trajectory. They are only dependent on the final size of the tree, specifically the final size of stem height, crown size, and heartwood area. 2) $S_{t_{R}}$ is dependent on the whole growth trajectory. This implies that the fitness proxy can be divided into a final tree size dependent part, $P_{t_{R}}/m_{t_{R}}$, and a growth path and time-dependent part, $S_{t_{R}}e^{-m_{t_{R}}t_{R}}$. As consequence the optimal growth strategy is to: 1) grow to final tree size with high net production to mortality ratio, *P*/*m* (as implied by case 2 in appendix C), 2) Grow along a trajectory with a high survival probability, and 3) reaching the final tree size as quickly as possible, i.e. minimize $t_{R}$. It is not possible to maximize all three goals at the same time, for example there exist a trade-off between $P_{t_{R}}$and $t_{R}$*.* A longer growth period can result in higher net productivity, but at the cost of lower survival probability.

Cited Literatures

Aseev, S., and Kryazhimskii, A. 2003. The Pontryagin maximum principle for infinite-horizon optimal controls. (April).

Bellman, R. 1954. The theory of dynamic programming. Bull. Am. Math. Soc. **60**(6): 503–515.

Halkin, H. 1974. Necessary Conditions for Optimal Control Problems with Infinite Horizons. Econometrica **42**(2): 267–272. doi:10.2307/1911976.

Kirk, D.E. 2004. Optimal control theory: an introduction. Dover Publications.

Perrin, N., and Sibly, R.M. 1993. Dynamic Models of Energy Allocation and Investment. Annu. Rev. Ecol. Syst. **24**(1): 379–410. doi:10.1146/annurev.es.24.110193.002115.

Pontryagin, L.S. 1962. Mathematical Theory of Optimal Processes. CRC Press.
